# Extracellular Vesicle Subproteome Differences among *Filifactor alocis* Clinical Isolates

**DOI:** 10.3390/microorganisms10091826

**Published:** 2022-09-13

**Authors:** Kai Bao, Rolf Claesson, Georgios N. Belibasakis, Jan Oscarsson

**Affiliations:** 1 Division of Oral Diseases, Department of Dental Medicine, Karolinska Institutet, 14104 Huddinge, Sweden; 2 Division of Oral Microbiology, Department of Odontology, Umeå University, 90187 Umeå, Sweden

**Keywords:** *Filifactor alocis*, oral infections, extracellular vesicles (EVs), predicted EV subproteome, proteomics

## Abstract

*Filifactor alocis* is a Gram-positive asaccharolytic, obligate anaerobic rod of the Firmicutes phylum, which has recently been implicated in oral infections. Extracellular vesicles (EVs) are crucial conveyors of microbial virulence in bacteria and archaea. Previously, in highly purified EVs from the *F. alocis* reference strain ATCC 35896 (CCUG 47790), 28 proteins were identified. The present study aimed to use label-free quantification proteomics in order to chart these EV proteins, in the reference strain, and in nine less-well-characterized clinical *F. alocis* isolates. In total, 25 of the EV proteins were identified and 24 were quantified. Sixteen of those were differentially expressed between the ten strains and the novel FtxA RTX toxin and one lipoprotein were among them. Consistent expression was observed among ribosomal proteins and proteins involved in L-arginine biosynthesis and type IV pilin, demonstrating a degree of EV protein expression preservation among strains. In terms of protein–protein interaction analysis, 21 functional associations were revealed between 19 EV proteins. Interestingly, FtxA did not display predicted interactions with any other EV protein. In conclusion, the present study charted 25 EV proteins in ten *F. alocis* strains. While most EV proteins were consistently identified among the strains, several of them were also differentially expressed, which justifies that there may be potential variations in the virulence potential among EVs of different *F. alocis* strains.

## 1. Introduction

It has been evident for decades that bacteria, archaea, and eukaryotes produce extracellular vesicles (EVs) during normal growth, which represent a universal mechanism to export proteins and other bacterial cargo to external targets [1,2]. EVs are released by both commensals and pathogens during normal growth, in vivo, and during infection of host cells in vitro. Vesicles from both Gram-negative and Gram-positive bacteria can carry out a number of offensive functions, including targeting concentrated virulence factors and inflammatory stimulants, such as LPS and peptidoglycan fragments, to host cells and tissues to manipulate the host immune response [2,3,4,5,6]. *Filifactor alocis* is a Gram-positive asaccharolytic, obligate anaerobic rod-shaped bacterium, belonging to the phylum Firmicutes. This organism was recently discovered in the oral microbiome via high-throughput DNA sequencing methodology and has, thereafter, been successfully cultivated in laboratories. *F. alocis* is considered an emerging oral pathogen with significant roles in the etiology of periodontitis [7,8] and, further, it is proposed to be a diagnostic indicator of periodontal disease [9,10]. Moreover, the species is implicated in additional oral diseases, including peri-implantitis [11] and endodontic [12,13] infections.

Intriguingly, *F. alocis* can paralyze neutrophils, the principal innate immune cell recruited to the periodontal pocket and extend their functional lifespan [14,15,16]. In particular, their capability of forming neutrophil extracellular traps (NETs), phagosome maturation, and reactive oxygen species (ROS) production were prevented, enhancing bacterial survival upon phagocytosis [17,18,19]. Hence, in contrast to many periodontopathogenic bacteria, *F. alocis* does not block phagocytosis by neutrophils [18,19]. Recently, the manipulation of human macrophages by *F. alocis* was demonstrated, which may further highlight the pathogenetic role of this bacterium in periodontal diseases [20]. Highly purified extracellular vesicles released by the *F. alocis* reference strain (ATCC 35896; CCUG 47790) were recently characterized regarding their proteomic content, using in-gel digestion and liquid chromatography-tandem mass spectrometry (LC-MS/MS) [21]. This revealed 28 proteins, including lipoproteins, autolysins, *F. alocis* complement inhibitor (FACIN), transporter- and metabolism-related proteins, and ribosomal proteins. Interestingly, the recently discovered repeats-in-toxins (RTX) protein family member, FtxA [22], was, according to its GenBank database definition, identified in the *F. alocis* EV proteome [21]. Whether FtxA or any of the other EV proteins might have played a role in the observed immunostimulatory effects of the vesicles on human monocytic and oral keratinocyte cell lines [21] and/or in the EV-mediated induction of osteoclastogenesis and systemic bone loss through Toll-like receptor 2 (TLR2) [23,24] is not presently clear. However, interestingly, as the osteoclastogenetic potency of *F. alocis* EVs was reduced upon treatment with lipoprotein lipase, lipoproteins may contribute to the systemic bone loss via TLR2 [24].

The present work aimed at providing further insights into the expression and functional conservation of the 28 EV proteins in nine additional, clinical *F. alocis* strains, by using our recently completed in-house proteomics database of these strains together [25]. Moreover, we charted these predicted EV proteins for their potential protein–protein interaction patterns and determined their levels of expression in the reference strain and in the nine less-well-characterized clinical *F. alocis* isolates.

## 2. Materials and Methods

### 2.1. Protein Identification and Label-Free Quantification

**Figure 1:**
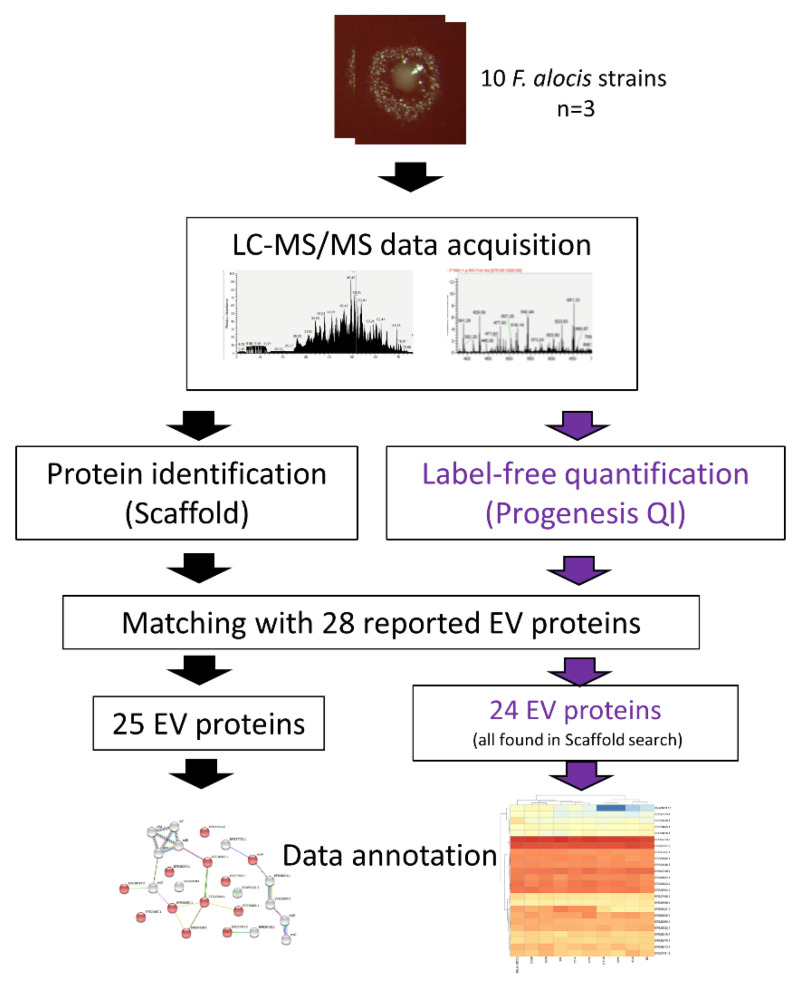
A schematic overview of the study design of the present work. The 28 EV proteins identified in the *F. alocis* reference strain ATCC 35896 and reported on previously [21] were assessed for predicted protein–protein interactions and expression levels in the reference strain and in nine additional, less-characterized *F. alocis* clinical isolates. A colony from *F. alocis* strain 624B-08U is shown at the top.

We recently obtained the full proteome of nine *F. alocis* clinical isolates and the reference strain ATCC 35896 (CCUG 47790), which had all been independently cultivated on agar for three days under the same conditions, using an Orbitrap Fusion Tribrid mass spectrometer interfaced to an Easy nano-flow HPLC system (Thermo Fisher Scientific, San Jose, CA, USA) [25]. To accurately characterize proteins in each strain, the protein identification and quantification were analyzed separately with two workflows (Figure 1). First, the proteins were identified by searching raw files individually using Mascot (version 2.51) against an in-house *F. alocis* database [25], with the following searching parameters: precursor tolerance: ±10 ppm; fragment ion tolerance: ±0.6 Da; instrument type: LTQ-ORBI-Default; enzyme: trypsin; maximum missed cleavages: 2; fixed medication: Carbamidomethyl (C); oxidation (M) and acetyl (Protein N-term). The search results were combined using Scaffold (version 4.2.1, Proteome software, Portland, OR, USA) at a cutoff of 3.0% FDR at the protein level (protFDR), minimal two peptides, and 1.0% FDR at the peptide level (pepFDR) for validation of the MS/MS-based peptide and protein identifications.

Thereafter, the protein abundances were quantified by aligning all raw files with their corresponding pooled sample as an alignment reference using Progenesis QI for Proteomics (Nonlinear Dynamics, Newcastle, UK). The aligned result was searched using Mascot (version 2.4.1, Matrix Science, London, UK) using the same parameters as described above for protein identification. Then, the spectrum reports of the search result were generated by Scaffold v4.0 (Proteome Software) with a threshold of protFDR of 1.0%, minimal one peptide and pepFDR of 0.5%, which was used by Progenesis QI for identifying the quantified proteins. Only proteins with at least two peptides identified were considered in the study.

### 2.2. Statistical Analysis

The protein quantification data derive from triplicate-normalized protein abundancies in each strain. The significance of differences for a specific protein between strains was calculated by ANOVA test in Progenesis QI. Proteins with an ANOVA test below 0.05 among all strains were considered as “truly regulated”.

### 2.3. Extraction of Proteomics Data Associated with the 28 F. alocis EV Proteins

The accession numbers of the 28 EV proteins identified previously in highly purified EVs from the reference strain CCUG 35896 [21] were used to retrieve their corresponding UniProt and GenBank identifiers from UniProt (https://www.uniprot.org/) (accessed on 19 March 2018). The list of retrieved identifiers was used to deduce the EV proteins from the full proteome data in both the Scaffold and Progenesis results.

### 2.4. Data Clustering and Heat Maps for Regulated Proteins

R software (R: A Language and Environment for Statistical Computing, R Development Core Team) and, in particular, the package pheatmap (https://cran.r-project.org/web/packages/pheatmap/index.html) (accessed on 24 October 2019) was used to generate unsupervised clustering analysis and heat maps of quantified proteins. Apparent outliers were removed from the study.

### 2.5. In Silico Functional Analysis for Identified EV Proteins

The prediction of protein–protein interactions and enriched analysis were performed using STRING (https://string-db.org/) (accessed on 21 May 2021). The interaction scores were calculated from experimental evidence as well as predictions based on knowledge gained from other organisms [26]. By using STRING, a network image based on confident scores (>0.15) between EV proteins was created. Among them, interactions with high confident scores (>0.9) were highlighted in circles. The EV proteins were subjected to STRING for “functional enrichment” against their whole genome. Enriched functions were recognized and ranked according to their FDR, using a hypergeometric distribution.

### 2.6. Image Procession

Images for figures were assembled using Microsoft PowerPoint (version 16; Microsoft, Redmond, WA, USA) and Adobe Photoshop (version CS6; Adobe, San Jose, CA, USA).

### 2.7. Ethical Considerations

All procedures were conducted in accordance with the guidelines of the local ethics committee at the Medical Faculty of Umeå University, which are in accordance with the Declaration of Helsinki (64th WMA General Assembly, Fortaleza, October 2013).

## 3. Results

### 3.1. Identification of the EV Proteins in Ten Different F. alocis Strains

Currently, many *F. alocis* proteins are not annotated with adequate functions in databases. Therefore, for example, none of the identified EV proteins in strain ATCC 35896 proteins were assigned with “extracellular vesicle [GO:1903561]” or “extracellular vesicle biogenesis [GO:0140112]” in the QuickGO database (https://www.ebi.ac.uk/QuickGO/) (accessed on 21st of May 2021). To investigate the potential functional conservation of this EV proteome in the ten *F. alocis* strains, we searched the list of 28 EV proteins reported by Kim et al. [21] and found that 25 of them matched our proteome list. Supplementary Table S1 lists all these proteins with their names in the UniProt database (https://www.uniprot.org) (accessed on 21 May 2021).

Figure 1 shows a schematic overview of the study design of the present work. Among the 25 identified EV proteins from our proteomics data, 19 were detected in all ten strains. On the other hand, the uncharacterized protein EFE27936.1, Thermonuclease (EFE28939.1), NLP/P60 domain protein (EFE27713.1), FtxA (ADW16141.1), Type IV pilin (EFE28478.1), and TRAP dicarboxylate transporter, DctP subunit (EFE29027.1) were only detected in two to eight strains, respectively (Table 1).

### 3.2. Predicted Protein–Protein Interactions of the EV Proteins

**Figure 2:**
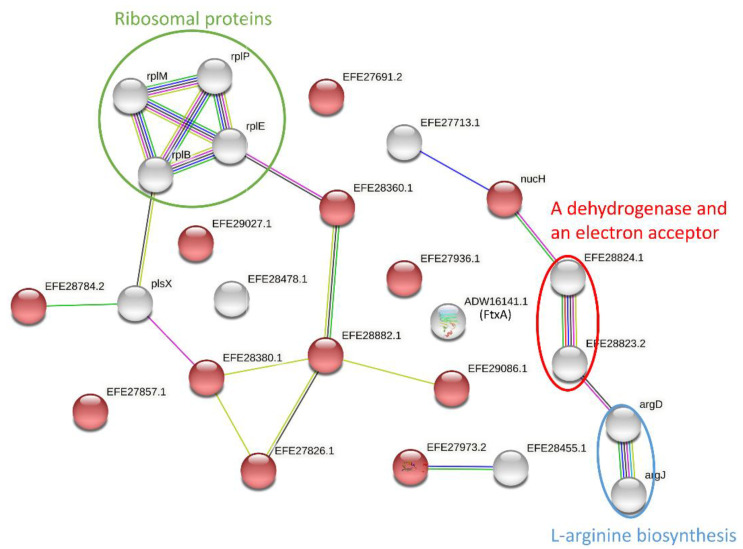
Predicted protein–protein interactions between EV proteins. Shown are networks established using STRING 10.5 based on the low confident score (0.15) of EV proteins (Supplementary Table S2). The interactions with high confident scores (0.9) are shown in circles. Proteins enriched for “signal” (UniProt: KW-0732) are indicated with red balls and the others with white. Colors of lines indicate different types of protein–protein interactions. Blue and purple lines indicate interaction determined from the curated database and experimental results, respectively. Green, red, and dark blue lines indicate predicted interactions determined from gene neighborhood, gene fusions, and gene co-occurrence, respectively. Yellow and black lines indicate interactions deduced from text mining and co-expression, respectively.

We also conducted a protein–protein interaction analysis using the STRING search tool and retrieved 21 functional associations between 19 proteins, albeit none involving FtxA (ADW16141.1). A number of interactions scored more than 0.95 (Figure 2, Supplementary Table S2). These included the interactions among four ribosomal proteins: RplB (EFE28922.1), RplE (EFE28913.1), RplM (EFE28170.1), and RplP (EFE28918.1). Moreover, strong functional interactions were retrieved between two enzymes involved in L-arginine biosynthesis (ArgD [EFE27651.2] and ArgJ [EFE27649.1]), as well as the interaction between the Butyryl-CoA dehydrogenase (EFE28824.1) and probably its electron acceptor, Electron transfer flavoprotein domain protein (EFE28823.2). Among the retrieved interactions, 13 pairs only exhibited low scores (<0.25). These analyses also showed that 12 EV proteins were enriched for the “signal” (UniProt: KW-0732) function, with a false discovery rate of 1.29 × 10^−7^ (Figure 2, Supplementary Table S2). The “signal” function indicates that these proteins contain a signal sequence, i.e., a peptide, usually present at the N-terminus of proteins that are destined to be either secreted or part of membrane components [27].

### 3.3. Quantification of the EV Proteins from Different F. alocis Strains

To further understand the expression of these EV proteins in the ten *F. alocis* strains, we performed label-free quantification using ProgenesisQI and quantified 24 of 25 of the detected EV proteins. The only exception was the uncharacterized protein EFE27936.1, which was only detected in the reference strain ATCC 35896 and in strain 149A-17U (Table 1) and, therefore, might be excluded by the label-free quantification algorithm of ProgensisQI during the peak picking. The ANOVA test showed that the expression of 16 of the EV proteins was significantly different among ten strains (*p* < 0.05) (Supplementary Table S3). In contrast, the expression levels of three of four ribosomal proteins, two enzymes involved in L-arginine biosynthesis, two hypothetical proteins, and the type IV pilin was consistent among all strains (Supplementary Table S3). We also found that the reference strain ATCC 35896 had the highest number (five, all significantly differently expressed among the ten strains) of the most abundant EV proteins (Table 2, Supplementary Table S3). On the other hand, strain 413B3-17U exhibited the highest number (five, four of which were significantly differently expressed) of the least abundant EV proteins (Table 2, Supplementary Table S3).

**Figure 3:**
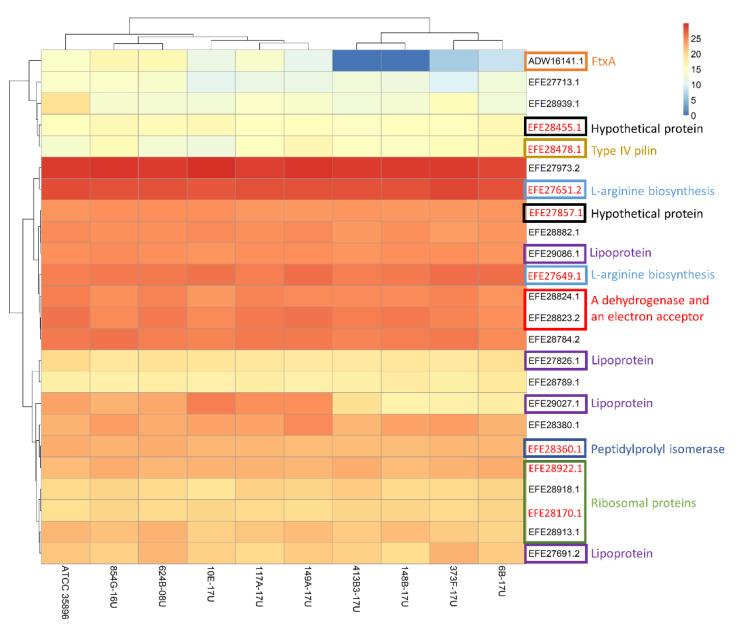
Heatmap of normalized abundance for EV proteins. The colors in the map display the mean value for log2-transformed normalized abundance value plus one for the individual proteins (represented by a single row) within each experimental group (represented by a single column). Expression values are shown as a color scale, with higher values represented by red and lower represented by blue. The EV proteins that were expressed at consistent levels (*p* > 0.05) among all strains are indicated with red text. Color-frame boxes and short explanations with the same color were given for proteins with consistent expressions as well as proteins with interaction confidence of more than 0.9 based on STRING analysis.

The log-2-transformed normalized abundances for all 24 quantified proteins are shown as a heatmap (Figure 3). Based on the unsupervised clustering, four *F. alocis* strains, i.e., 6B-17U, 373F-17U, 148B-17U, and 413B3-17U, were found to have a similar pattern of EV protein expression, including lack of expression of FtxA (ADW16141.1). This pattern of EV protein expression was more distant to the reference strain ATCC 35896 than the other clinical isolates (Figure 3). Moreover, the heatmap showed that the four ribosomal proteins were expressed at similar abundances. This was observed also for Butyryl-CoA dehydrogenase (EFE28824.1) and Electron transfer flavoprotein domain protein (EFE28823.2). Although three of four quantified lipoproteins were expressed at a similar abundance in all strains, the expression level of TRAP dicarboxylate transporter, DctP subunit (EFE29027.1), was clearly higher in the FtxA-expressing strains than in those lacking expression of FtxA.

## 4. Discussion

*F. alocis* is a rather recently discovered species, with relevance to several oral infections, notably periodontitis [7,8,9]. The reference *F. alocis* strain ATCC 35896 and nine additional, less-well-characterized clinical strains were recently proteomically analyzed using Scaffold [25]. Moreover, they were characterized with regards to the newly discovered RTX toxin family member, FtxA [22], a property that renders this organism as one of few known exotoxin-producing oral pathogens [28]. In the present study, particular focus was placed on the proteomic analysis of EV proteins, i.e., the EV subproteome earlier defined by Kim et al. [21]. Hence, we analyzed the proteins in this EV subproteome, rather than EVs per se, nor purified them. Since limited information exists in this field, our data are going to be discussed (a) in relation to the role of the clusters of proteins identified in the database, as already registered in other species, and (b) in comparison to already databased *F. alocis* vesicle proteins. In proteomics studies, GO term identification and enrichment analysis are procedures frequently used to chart a subset of proteins (i.e., subproteome) based on their GO assignments in the full proteome [29]. Evidently, however, in many cases, functional annotations of *F. alocis* proteins in the QuickGO database were not sufficient during the present study, which made it difficult to rely on GO terms to distinguish the EV proteins from our identified protein list. As an alternative approach, we, therefore, narrowed down the available data on the functions of the 28 EV proteins recently identified in highly purified vesicles from the *F. alocis* reference strain ATCC 35896 [21], which can be considered as a representative proteome of EVs from this species and, hence, suitable to be used as a basis for comparisons of expression levels of vesicle proteins in the ten assessed strains. Our main interest in the present work was, thus, to compare the abundances of these 28 EV proteins among the different strains, which could be used as a foundation to elucidate the potential virulence properties of their EVs in future assessments. An apparent technical disadvantage of using the full proteome context to compare with purified EVs is the possibility that EV proteins might not be sufficiently identified due to their relatively low abundances, which motivates approaches with subproteomics on bacteria [30]. Therefore, our present analysis was designed on the basis of the recently delineated full proteomes of each strain [25], to which the abundances of the 28 EV proteins in this subproteome could be normalized. Among these 28 proteins, 25 matched with the proteome list yielded in the present study, of which 19 were detected in all ten *F. alocis* strains, whereas the remaining 6 were detected only in some of the strains. The present observation that FtxA was detected only in six of the ten *F. alocis* strains is consistent with the absence of the *ftxA* gene in the other strains [22]. We, nevertheless, concluded that there is good agreement between the findings of the earlier study [21] and the present work. A protein–protein interaction analysis using STRING retrieved 21 functional associations between 19 proteins. While 13 paired interactions yielded low scores, it is worth noting that the highest interaction scores were noted among the four ribosomal proteins and the two enzymes involved in L-arginine biosynthesis. This functional observation aligns well with the finding that those EV proteins are more consistently expressed, without abundance variations, among all strains. Interestingly, FtxA did not display interactions in STRING with any of the designated EV proteins. This is consistent with an apparent absence of the other proteins encoded by the *ftx* operon, FtxC, and FtxD in the EVs [21] and with observations with other RTX toxins, such as the *Aggregatibacter actinomycetemcomitans* leukotoxin (LtxA), which was present in OMVs released by that species, whereas LtxC, LtxB, and LtxD were absent [4,31]. The lack of interactions in STRING indicates that FtxA may exist in a post-translational form in the EVs, ready to be released extracellularly.

Among the 25 identified EV proteins in the present work, 24 were quantified and 16 of those proved to be expressed at significantly different levels among the studied ten strains. Consistent expression was observed among ribosomal proteins and ones involved in L-arginine biosynthesis and type IV pilin, which demonstrates a degree of preserved EV functions among the strains. Interestingly, the reference strain ATCC 35896, which is also the most studied one in the literature [7,9], showed the highest number of most abundant EV proteins among the ten strains. Four of the clinical *F. alocis* isolates showed a similar and distinct pattern of EV protein expression abundances (including the ribosomal proteins), which was more distant to the reference strain ATCC 35896 than the other strains. Interestingly, these four strains were the ones lacking the *ftxA* gene [22], suggesting that this putative exotoxin may account for the variation in the virulence and EV-associated pathogenic mechanisms of *F. alocis* strains. Moreover, as the four strains lacking the *ftxA* gene, in contrast to the other, exhibited very low expression of one of the EV lipoproteins (EFE29027.1), it is possible that the EVs of these strains may be less efficient in inducing systemic bone loss via TLR2 [24]. It is not known whether there is virulence variability between the ten *F. alocis* strains assessed in the present study. They were all isolated from various oral infections, including periodontitis, peri-implantitis, acute necrotizing ulcerative gingivitis (ANUG), and endodontic infections [22]. Nevertheless, according to our previous observations, strain 624B-08U was found to be almost totally dominant (>90% out of the total viable count) in the analyzed clinical sample from which it was isolated (ANUG) [22]. Hence, it could be speculated that this might represent a strain with enhanced virulence. Our present observation that this strain exhibited the second-highest levels of FtxA among the ten tested strains may suggest that FtxA, in some cases, can contribute to domination in specific niches in the oral cavity. This remains to be investigated. Proteome differences among two different *F. alocis* strains were initially identified based on early proteomics investigations [32] and, recently, ten strains were assessed and compared using label-free quantification [25]. While those analyses did not take into consideration EV-localized proteins per se, several of the identified proteins were indeed associated with the outer membrane in the organism. In addition, more cell wall anchoring proteins were identified in a virulent clinical *F. alocis* isolate, D-62D, than in the reference strain ATCC 35896 [32].

Our present study charted the proteins of the EV subproteome in the *F. alocis* reference strain ATCC 35896, in ten strains altogether. Taken together, there is functional conservation among the most consistently expressed EV proteins, which involve ribosomal and L-arginine biosynthetic activity. The reference strain of *F. alocis*, ATCC 35896, appears to have the highest abundances in EV proteins. The remaining variation in EV protein presence and abundance among strains could well account for variations in the virulence among the strains. Taken together, the present work may serve as a basis for future functional analyses assessing *F. alocis* vesicles, including their effect on host cells.

## Figures and Tables

**Table 1:** Detection of the EV proteins in the ten *F. alocis* strains. All EV proteins detected by label free in each of the ten strains, respectively, are listed.

Accession	ATCC 35896	854G-16U	117A-17U	149A-17U	624B-08U	10E-17U	373F-17U	6B-17U	413B3-17U	148B-17U	Detection Ratio
EFE27936.1	Yes	No	No	Yes	No	No	No	No	No	No	2 of 10
EFE28939.1	Yes	No	No	No	No	No	Yes	No	No	No	2 of 10
EFE27713.1	No	No	No	No	Yes	No	No	Yes	Yes	Yes	4 of 10
ADW16141.1	Yes	Yes	Yes	Yes	Yes	Yes	No	No	No	No	6 of 10
EFE28478.1	Yes	Yes	Yes	Yes	No	No	Yes	Yes	Yes	Yes	8 of 10
EFE29027.1	Yes	Yes	Yes	Yes	Yes	Yes	No	Yes	Yes	No	8 of 10
EFE27649.1	Yes	Yes	Yes	Yes	Yes	Yes	Yes	Yes	Yes	Yes	10 of 10
EFE27651.2	Yes	Yes	Yes	Yes	Yes	Yes	Yes	Yes	Yes	Yes	10 of 10
EFE27691.2	Yes	Yes	Yes	Yes	Yes	Yes	Yes	Yes	Yes	Yes	10 of 10
EFE27826.1	Yes	Yes	Yes	Yes	Yes	Yes	Yes	Yes	Yes	Yes	10 of 10
EFE27857.1	Yes	Yes	Yes	Yes	Yes	Yes	Yes	Yes	Yes	Yes	10 of 10
EFE27973.2	Yes	Yes	Yes	Yes	Yes	Yes	Yes	Yes	Yes	Yes	10 of 10
EFE28170.1	Yes	Yes	Yes	Yes	Yes	Yes	Yes	Yes	Yes	Yes	10 of 10
EFE28360.1	Yes	Yes	Yes	Yes	Yes	Yes	Yes	Yes	Yes	Yes	10 of 10
EFE28380.1	Yes	Yes	Yes	Yes	Yes	Yes	Yes	Yes	Yes	Yes	10 of 10
EFE28455.1	Yes	Yes	Yes	Yes	Yes	Yes	Yes	Yes	Yes	Yes	10 of 10
EFE28784.2	Yes	Yes	Yes	Yes	Yes	Yes	Yes	Yes	Yes	Yes	10 of 10
EFE28789.1	Yes	Yes	Yes	Yes	Yes	Yes	Yes	Yes	Yes	Yes	10 of 10
EFE28823.2	Yes	Yes	Yes	Yes	Yes	Yes	Yes	Yes	Yes	Yes	10 of 10
EFE28824.1	Yes	Yes	Yes	Yes	Yes	Yes	Yes	Yes	Yes	Yes	10 of 10
EFE28882.1	Yes	Yes	Yes	Yes	Yes	Yes	Yes	Yes	Yes	Yes	10 of 10
EFE28913.1	Yes	Yes	Yes	Yes	Yes	Yes	Yes	Yes	Yes	Yes	10 of 10
EFE28918.1	Yes	Yes	Yes	Yes	Yes	Yes	Yes	Yes	Yes	Yes	10 of 10
EFE28922.1	Yes	Yes	Yes	Yes	Yes	Yes	Yes	Yes	Yes	Yes	10 of 10
EFE29086.1	Yes	Yes	Yes	Yes	Yes	Yes	Yes	Yes	Yes	Yes	10 of 10

**Table 2:** EV protein abundancies in the ten *F. alocis* strains. The numbers of most or least abundant EV proteins in each *F. alocis* strain, as determined by label-free quantification proteomics, are indicated.

*F. alocis* Strain	All 24 Proteins		16 Regulated Proteins	
	Most Abundant	Least Abundant	Most Abundant	Least Abundant
10E-17U	3	4	2	2
117A-17U	0	1	0	0
148B-17U	0	3	0	2
149A-17U	3	1	2	0
373F-17U	3	3	1	3
413B3-17U	1	5	1	4
624B-08U	3	0	1	0
6B-17U	1	4	1	4
854G-16U	5	1	3	1
ATCC 35896	5	2	5	0

## Data Availability

The in-house database and mass spectrometry *F. alocis* proteomics data were earlier deposited to the ProteomeXchange Consortium via the PRIDE partner repository with the dataset identifier PXD026971 [25]. The authors declare that all data supporting the findings of the present study are available within the article and its Supplementary Information or upon request from the corresponding author.

## Supplementary Materials

The following supporting information can be downloaded at: https://www.mdpi.com/article/10.3390/microorganisms10091826/s1, Supplementary Table S1. Identified *F. alocis* EV proteins using Scaffold. Supplementary Table S2. STRING analysis results for protein–protein interactions of the *F. alocis* EV proteins. Supplementary Table S3. Quantified *F. alocis* EV proteins using Progenesis.
